# Numerical study on performance of a vertical axis wind rotor with S-shaped blades

**DOI:** 10.1371/journal.pone.0322953

**Published:** 2025-05-27

**Authors:** Lei Song, Ji Wang, Shibo Wang, Zongxiao Yang, Jianxin Su

**Affiliations:** 1 College of Mechatronics Engineering, Henan University of Science and Technology, Luoyang, Henan, China; 2 Collaborative Innovation Center of Henan Province for High-End Bearing, Henan University of Science and Technology, Luoyang, Henan, China; 3 College of Vehicle and Traffic Engineering, Henan University of Science and Technology, Luoyang, Henan, China; Yanbu Industrial College, EGYPT

## Abstract

Traditional Savonius wind rotor has simple structure and can operate in any wind direction. However, its aerodynamic efficiency is lower than other types. A novel S-shaped wind rotor with three blades is proposed in order to improve the aerodynamic performance. The blade of the rotor is composed of two opposing convex circular arcs and its shape likes an ‘S’. The flow characteristics of the rotor are studied and analyzed by computational fluid dynamics (CFD) numerical simulation method. The steady and transient performances are studied using SST k-ω model and sliding mesh method, and are compared with that of traditional Savonius rotors. The results show that the average static torque coefficient of the rotor is 0.291, which is higher than the 0.222 of the Savonius rotor. The static vibration amplitude of the rotor is 0.375, which is lower than 0.709 of the Savonius rotor. The maximum power coefficient of the rotor is 0.228, which is also higher than the 0.226 of the Savonius rotor. The dynamic vibration amplitude of the novel rotor is 0.183, which is lower than the 0.492 of the Savonius rotor. The flow field analyses show that structure of the S-shaped blades can smooth the flow field and reduce the blocking effect in the overlap area. The study indicates that the proposed navel rotor can not only overcome the problems of sharp change in the internal flow field of traditional Savonius rotors, but also provide better operating stability and higher wind energy utilization.

## 1. Introduction

With the shortage of traditional fossil energy and the increasingly prominent environmental problems, clean energy has become an important research field in countries around the world at present. Wind energy is a renewable energy without pollution in nature world. It has inexhaustible reserve and has been taken more and more attentions by the whole world. According to relationship between rotating axis of wind rotor and direction of wind, there are two primary categories of modern wind rotors classified as horizontal axis wind turbines (HAWTs) and vertical axis wind turbines (VAWTs). The wind energy utilization of HAWTs is usually higher than that of VAWTs. However, VAWTs have their own advantages. They can run at any direction of wind, work without yaw system and have low manufacturing costs [1,2].

VAWTs are generally divided into two broad categories: lift-driven type and drag-driven type. The wind energy utilization of the drag-driven rotors is lower than that of the lift-driven rotors, but they have good self-starting performance and can work under low speeds [3,4]. The typical drag-driven rotor is Savonius rotor. Worasinchai et al. studied the start-up performance of savonius wind turbines [5]. The results showed that the maximum torque of the three-blade rotor is lower than that of the two-blade rotor, but the torque distribution is relatively smooth. Both Han et al. and Nasef et al. studied the static and dynamic performance of Savonius wind turbines [6,7]. The results showed that the performance of wind turbines is greatly affected by the geometrical structure and overlap ratio of blades. The research results of scholars show that the overlap area composed by blades of the traditional Savonius rotors often produces blocking effect, and eddy currents are formed near the sharp change of the airflow. It leads to energy loss and reduces its performance.

In order to improve the performance of Savonius wind rotors, scholars have conducted a lot of research. Laws et al. built a novel type of blade by thickening the middle part of the blade of traditional Savonius wind turbine and shrinking slightly flat at both ends [8]. Compared with Savonius wind turbine, the maximum power coefficient increased by 28.12%. Aranizadeh et al. studied the control strategy of wind resource conversion devices such as horizontal axis wind turbines and Savonius wind turbines [9,10]. Ozbak et al proposed a wind speed monitoring method to artificially improve the output power of wind turbines [11]. Thomai et al. optimized the Bach wind turbine and helically processed the Bach blade structure in space, which significantly improved the overall performance of the optimized wind turbine [12]. It can be seen that the structure of the rotor is closely related to its aerodynamic performance. Appropriate change of wind turbine structure can significantly improve the aerodynamic performance of wind rotor.

Blade profile plays a crucial role in rotor performance. Therefore, many researchers consider to improve the aerodynamic performance of the wind rotor by changing the shape structure of the blade. Wang et al. studied the influence of blade leading edge defects on aerodynamic characteristics and flow characteristics of wind turbines [13]. Abdelghafar et al. built a bionic blade shape of Savonius wind turbine inspired by sand dunes to improve the aerodynamic performance of wind turbines [14]. Sobczak et al and Bouzaher et al proposed deformable blades that can change their form during the turbine rotation, and it could increase a positive torque of the advancing blade and to decrease a negative torque of the returning blade [15–17]. Tata et al. studied seven types of two-blade wind turbines with different blade structures, and the results showed that wind turbines with elliptical blades had the best aerodynamic performance [18]. These research results show that the performance of wind rotors can be significantly improved by changing the blade structure in different ways. However, considering the complexity of wind rotor blade structure and manufacturing costs, further explore of novel blade structure is also needed [19]. Mu et al and Mendoza et al obtained Twisted Blades by twisting Savonius blade structure [20,21]. The research results showed that wind turbine with twisted Blades can improve the start-up performance and output performance of wind turbines. Norouztabar et al. [22] built an improved triple-blade wind turbine, which improved the performance of wind turbines. Mari et al. [23] proposed a spline rotor to improve the aerodynamic performance of the wind turbines by improving the structure of Savonius wind turbines. Talukdar et al. combined semi-circular blade and concave elliptical blade to build a circular elliptic blade [24]. The research results showed that the blade has greater local torque around the blade than Savonius blade. It can be seen that the combination method can construct an efficient wind rotor structure by simple configuration, so as to improve the aerodynamic characteristics of wind rotors. Pan et al. combined Savonius wind turbine and Darrieus wind turbine to build a hybrid wind turbine to improve the aerodynamic characteristics of wind turbines [25]. Etemadeasl et al. combined two Savonius rotors to build the Counter-Rotating Savonius Rotors [26]. The results showed that the aerodynamic performance of rotors has been significantly improved. Hossain et al and Tian et al combined two wind turbine blades to build an S-shaped Savonius rotor without overlap ratio [27,28]. The research results showed that S-shaped blades could improve the output performance and starting torque of the wind rotor. This shows that the performance of wind rotors can be improved without increasing the complexity of blades, and the research results of scholars provide ideas for the construction of novel wind rotor structures.

In the study of wind rotor performance optimization, the influence of structural parameters on aerodynamic performance is also studied. Chabane et al. studied the effects of blade number on tip speed ratio, torque, wind turbine power and torque force of vertical wind turbines [29]. Zhang et al. [30] studied the influence of blade thickness and camber on the power coefficient performance of wind turbines. The results showed that with the increase of the thickness, the power coefficient showed a trend of first increasing and then decreasing. Hosseini et al. studied the influence of overlap ratio on aerodynamic performance of wind turbines and found the optimal overlap ratio range of Blades SR3345 and SR5050 [31]. Abdelaziz et al. demonstrated that adjusting the overlap ratio of wind turbines can reduce the wake generated in the overlap area and improve the output performance [32]. The research results of these scholars all show that there is a close relationship between structural parameters such as blade number and overlap ratio of wind rotors and aerodynamic performance. The effect of structural parameters on wind rotor performance is very significant. Therefore, it is necessary to consider the influence of important parameters such as blade number and overlap ratio when blade structure is constructed.

At present, the research methods of wind rotor performance are mainly based on wind tunnel test and numerical simulation. Wind tunnel test is the most effective method to study the performance of the wind turbine. Al-Gburi et al. studied the performance of wind turbines through a combination of experimental research and numerical analysis [33]. Wind tunnel test can obtain real performance data of wind rotors. However, its economic and time costs are too high. In recent years, with the continuous development of computer level, computational fluid dynamics (CFD) has become the main means to study the aerodynamic performance of wind rotors. Aboujaoude et al. aimed to aerodynamically optimize the deflector shape through transient 3D CFD simulations using sliding mesh techniques [34]. Maalouly et al and Fatahian et al used unstructured grid and SST k-ω model for numerical simulation of wind turbines [35,36]. Due to the advantages of numerical simulation such as low cost and excellent visualization effect, more and more scholars begin to study the aerodynamic characteristics of wind rotors by CFD technology. Alom et al. used six different turbulence models to conduct two-dimensional unsteady numerical simulation of Savonius rotor [37]. The results showed that the SST k-ω model could better predict the flow separation and flow characteristics of Savonius rotor. The SST k-ω model often has higher accuracy and faster calculation speed in numerical simulation.

Based on thinking and research results of scholars, a novel structure of VAWT rotor is proposed to improve the performance and wind energy utilization. The rotor is composed by three blades which each structure is shaped as an ‘S’. The flow characteristics of the rotor are studied and analyzed by computational fluid dynamics (CFD) numerical simulation method. The steady and transient performances are studied using SST k-ω model and sliding mesh method, and are compared with that of traditional Savonius rotors.

## 2. Geometry configuration

### 2.1. Geometry of the wind rotor

**Fig 1:**
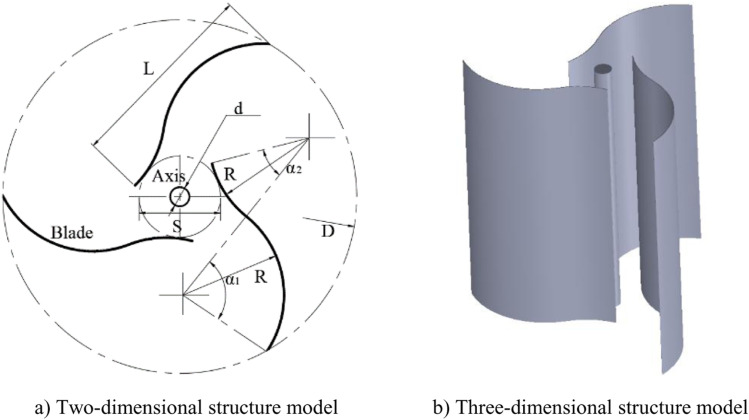
The structure model of the S-shaped rotor. a) Two-dimensional structure model. b) Three-dimensional structure model.

Two important factors causing low wind energy utilization of the two-blade Savonius rotors are the wind resistance of the rotating blade and the sharp change of the airflow in the overlap area [34]. The configuration structure with three-blade can improve the characteristics of the internal flow field of the rotor. The wind energy utilization of the three-blade Savonius rotor is lower than that of the two-blade Savonius rotor. In order to guide the airflow passing through the overlap area smoothly to reduce the blocking effect and improve aerodynamic performance, the rotor blade is designed by composing of two convex circular arcs which opposite each other. Therefore, the blade is a combination of two arcs. Its structural complexity does not increase much and shape looks like an ‘S’. Lengths of the two arcs are different. Radiuses of them are set to be equal with each other in order to simplify the structure. The overall structure of the rotor is composed of three S-shaped blades and the blades are arranged uniformly around the central axis, as shown in Fig 1a. The three-dimensional structure of the three-blade S-shaped rotor is shown in Fig 1b.

In Fig 1, *R* is radius of the arcs, *α*_*1*_ and *α*_*2*_ are angles of the two arcs respectively, *L* is linear length of a blade, *D* is diameter of the rotor and *S* is diameter of inscribed circle which is surrounded by three small arcs of blades closing to central axis. Similar to Savonius rotor, the inner space surrounded by three small arcs is defined as overlap area. Function of this area is to allow airflow through it smoothly to leeward side after it has driven a blade. To reduce the sharp change of airflow through the overlap area, number of blades is considered increasing to reduce the eddy current, and the three-blade scheme is adopted initially. Two segments of arcs in a blade are linked smoothly and arranged by facing forward the convex shapes with each other. The main function of concave face of the big arc is to accept wind energy and support impact of airflow, and its curvature radius has an important effect on the rotor performance. The inner tip of the small arc orients toward the convex face of the adjacent blade. The main function is to favorably guide airflow passing through overlap area to act on convex face of the next blade to improve streamline and then to drive the rotor. The total central angles of two arcs are initially selected to be less than 180° to reduce the shielding effect between blades.

**Fig 2:**
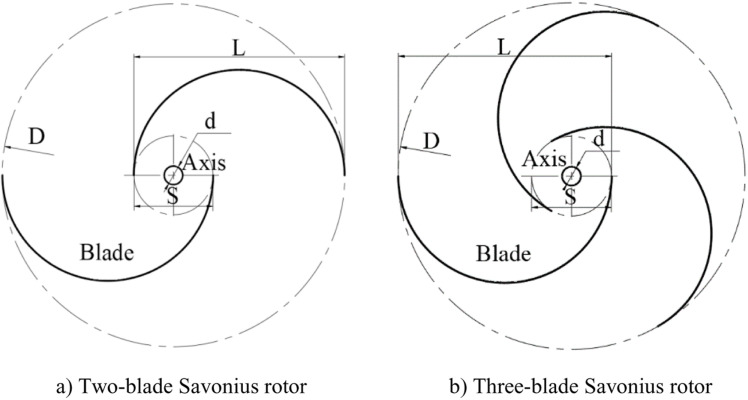
Geometrical structures of the Savonius rotors. a) Two-blade Savonius rotor. b) Three-blade Savonius rotor.

Two traditional Savonius rotor models with two blades and three blade are selected for performance comparison with the novel rotor in this paper, and their structures are shown in Fig 2a, b respectively. The research results of Al-Kayiem et al. showed that the Savonius rotor obtain a higher wind energy utilization when the overlap ratio is 0.15 [38]. Therefore, the overlap ratio of the two Savonius rotors selected for comparative analysis in this paper is set as the optimal value of 0.15 and the structural parameters of the rotor are calculated accordingly.

The main structure parameters of the three wind rotors are shown in Table 1.

**Table 1:** Main parameters of the three wind rotors.

	S-shaped rotor	Two-blade Savonius rotor	Three-blade Savonius rotor
Number of blades	3	2	3
Diameter of rotor(mm)	700	700	700
Height of rotor(mm)	700	700	700
Diameter of central axis(mm)	45	45	45
Overlap ratio	0.166	0.15	0.15
Radius of curvature of blade(mm)	200	212.5	212.5
α1 (°)	85	–	–
α2(°)	36	–	–

### 2.2. Aerodynamics of Savonius rotor

Performance parameters are important indicators that can be used to evaluate the performance of rotors. The main parameters of Savonius rotor includes overlap ratio (*OL*), tip speed ratio (*TSR*), power coefficient (*C*_*p*_) and torque coefficient (*C*_*M*_).

Overlap ratio (*OL*) can be defined as ratio of diameter of overlap area and that of wind rotor. According to the research results of Hosseini et al., the value of overlap ratio affects the aerodynamic performance of wind rotors [31]. When a central axis is included, the *OL* can be modified as shown in Eq 1.


$$OL=\frac{S-d}{D}$$ (1)

Where *S* is the inscribed circle diameter of the inner edge of each blade of wind rotor, *D* is diameter of wind rotor and *d* is diameter of central axis of the wind rotor.

Tip speed ratio (*TSR*) is an important dimensionless parameter to describe rotor performance. It describes the ratio relationship between the linear velocity of the blade tip and the wind speed, and can be calculated by Eq 2.


$$\lambda =\frac{R\omega }{v}$$ (2)

Where *ω* is angular velocity of the rotor, *R* is radius of the rotor and *v* is wind speed.

Power coefficient *C*_*p*_ represents the fraction of extracted power from the total power of airflow which runs through the projected area of rotors in the flow direction. Torque coefficient *C*_*M*_ is an important index to evaluate the performance of rotors. Fatahian et al. used *C*_*p*_ and *C*_*M*_ parameters to demonstrate the performance of rotors [36]. *C*_*p*_与*C*_*M*_ can be obtained by Eqs 3 and 4.


$$C_{p}=\frac{P}{0.5\rho Av^{3}}$$ (3)

$$C_{M}=\frac{M}{0.5\rho Av^{2}R}$$ (4)

Where *P* is the power of wind rotor, *M* is the torque developed around the axis of the wind rotor, *ρ* is the air density, *A* is the swept area, *R* is the radius of wind rotor.

## 3. Numerical method

### 3.1. Computational domains and grid generation

**Fig 3:**
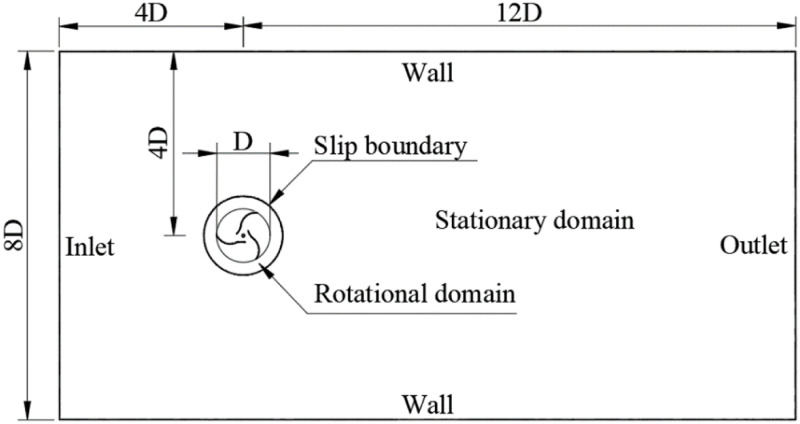
Computational domain and boundary conditions.

The simulation model of the rotor is simplified to two-dimensional flow field because the cross-section shape of the rotor in the height direction is the same. The simulation computational domain model is shown in Fig 3. Rotating domain is enlarged in Fig 3 to show the internal situation better. The overall domain is defined as that the length between inlet which is located at left side and center of the rotor is 4 times as much as the rotor diameter. The length between center of the rotor and outlet is 12 times as much as the rotor diameter and the height of the domain is 8 times of the rotor diameter.

**Fig 4:**
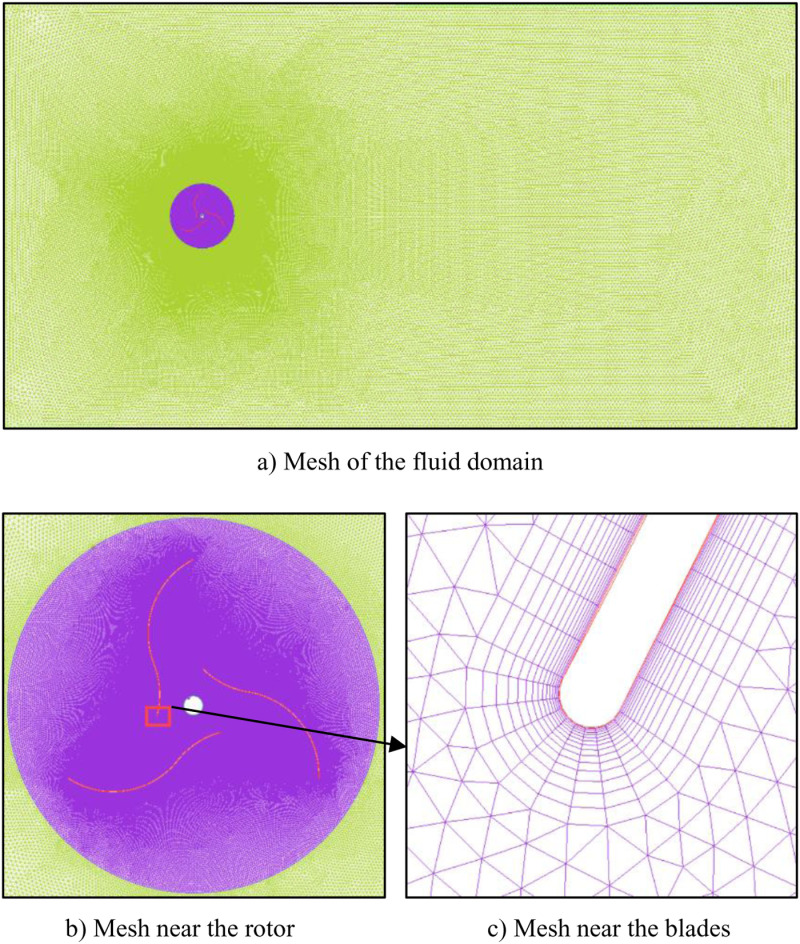
Mesh of the model. a) Mesh of the fluid domain. b) Mesh near the rotor. c) Mesh near the blades.

**Fig 5:**
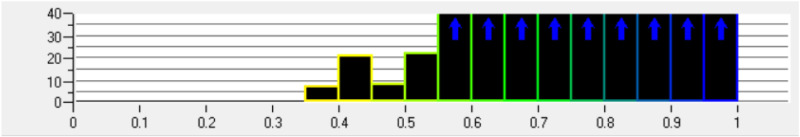
Meshes of aspect ratio.

**Fig 6:**
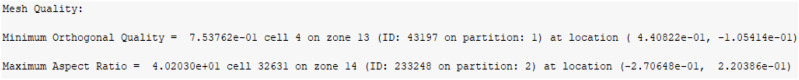
Meshes quality.

The computational domain is divided by unstructured grid. In order to capture the flow of boundary layer on the airfoil surface and improve the accuracy of simulation results, boundary layer mesh is generated on the surface of the blade airfoil and the surface of the rotation axis, as shown in Table 2. The mesh division results of the simulation model are shown in Fig 4. The mesh division of the global computational domain and rotation domain are shown in Figs 4a, b. The mesh division at the end of the blade is displayed in Fig 4c. The aspect ratio of the divided mesh is shown in Fig 5. It can be seen that the number of meshes with aspect ratio less than 0.55 is 46. The mesh quality after post-smoothing is shown in Fig 6.

**Table 2:** Setting of boundary layer mesh parameter.

	First layer thickness(mm)	Height radio	Num layers
Axis	0.03	1.2	17
Blades	0.03	1.2	17

Sliding Mesh (SM) method is widely used in engineering for good simplicity and applicability [39]. The circle in Fig 3 is defined as an interface between the rotational domain and the stational domain. The fluid around rotor is defined as moving mesh with rotational speed, and blades are defined as moving wall with rotational motion. In the processes of SM method for the rotor, *SST k-ω* viscous model is used. SIMPLE scheme of pressure-velocity coupling and second order upwind in spatial discretization are selected to solve the simulation because of its higher calculating accuracy. Inlet is defined as velocity-inlet and outlet is as outflow. The rotor is set to revolve counterclockwise for positive driving force. Table 3 shows the solver Settings for the rotor.

**Table 3:** Setting of the solver.

Domain	Setting
Turbulence model	SST k-ω
Pressure-velocity coupling scheme	SIMPLE
Discretization order	2nd order
Inlet	Velocity-inlet
Outlet	Outflow
Wall	Symmetry
CFD approach	Incompressible unsteady Reynolds- averaged Naiver-Stokes
Time step	1°
Rotor direction	Counterclockwise

The turbulent flow of wind rotor is simulated and analyzed through *SST k-ω* viscous model. The transport equations are as Eqs 5 and 6.


$$\mathrm{\backslash [}\frac{\partial }{\partial t}\left(\rho k\right)+\frac{\partial }{\partial \chi_{i}}\left(\rho k{\text{u}}_{i}\right)\text{=}\frac{\partial }{\partial \chi_{i}}\left[\Gamma_{k}\frac{\partial k}{\partial \chi_{j}}\right]+G_{k}-Y_{k}+S_{k}\mathrm{\backslash ]}$$ (5)

$$\mathrm{\backslash [}\frac{\partial }{\partial t}\left(\rho \omega \right)+\frac{\partial }{\partial \chi_{i}}\left(\rho \omega u_{i}\right)\text{=}\frac{\partial }{\partial \chi_{i}}\left[\Gamma_{\omega }\frac{\partial \omega }{\partial \chi_{j}}\right]+G_{\omega }-Y_{\omega }+D_{\omega }+S_{\omega }\mathrm{\backslash ]}$$ (6)

Where *G*_*k*_ is the turbulent kinetic energy term generated by laminar velocity gradient,${\;\;\Gamma \;\;}_{\text{k}}$ and ${\;\;\Gamma \;\;}_{\;\;\omega \;\;}$ are the effective diffusivity of *k* and *ω*, *Y*_ω_ and *Y*_*k*_ are turbulence due to diffusion; *D* is the orthogonal divergent term.

### 3.2. Numerical method validation

#### 3.2.1. Grid independence verification.

**Fig 7:**
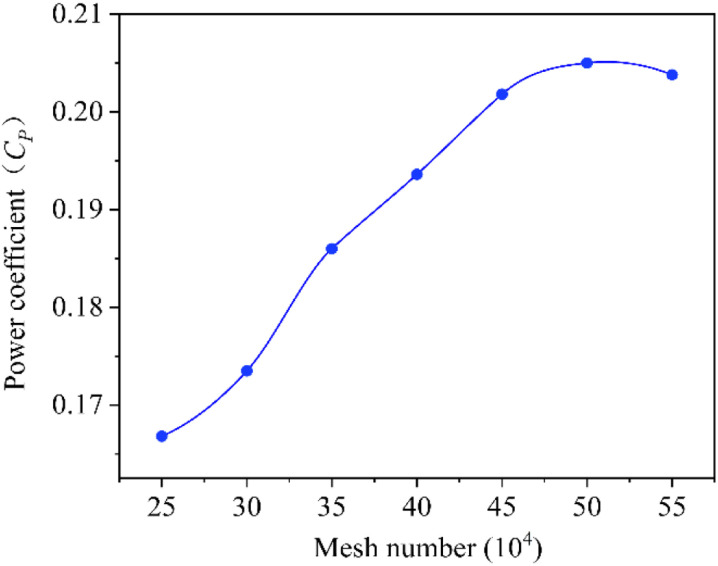
Power coefficients under different grid numbers.

The Grid Independent Limit (GIL) is studied to consider independence between number of grids and simulation results of the rotors. Seven samples are used for the calculations and the power coefficient of the rotor is taken as the parameter for which GIL is determined. Each level is solved in Fluent module of Software Ansys 2022. They are set to the same of input parameters with 10m/s of wind speed and 0.6 of *TSR*. The simulation is set to transient time model and the time step is set to the time needed for the rotor to perform an azimuthal rotation of 1º. The grid independence analysis of the rotor with various samples is shown in Table 4 and Fig 7. When number of elements is lower than level of 450,000, simulation result of power coefficient is susceptible to it. While number of elements exceeds this level, change of power coefficient value is very small. Therefore, a level of about 450,000 elements for the mesh is considered and set for final simulation.

**Table 4:** Power coefficients under different mesh numbers.

Mesh number	Power coefficient
248652	0.1668
302824	0.1735
349621	0.1908
399524	0.1936
451852	0.201
503527	0.205
548525	0.203

#### 3.2.2. Time independent verification.

**Fig 8:**
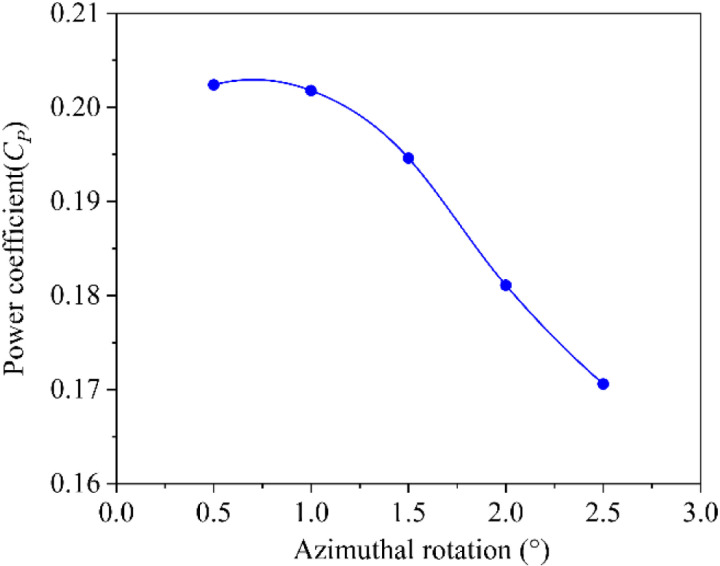
Power coefficients under different azimuthal rotations.

The accuracy of the calculation results is closely related to the time step size in the transient numerical simulation. Five different time steps are set respectively to validate time independence. The angular intervals are 0.5°, 1°, 1.5°, 2°, and 2.5° in each time step. Taking the power coefficient of the rotor as the comparison parameter, numerical simulation is carried out under the same working conditions and the results are shown in Fig 8. It can be seen that the power coefficient value changes little when the angular interval is less than 1°. Therefore, 1° is selected as time step to ensure the accuracy of numerical simulation calculation.

#### 3.2.3. Validation of numerical model.

**Fig 9:**
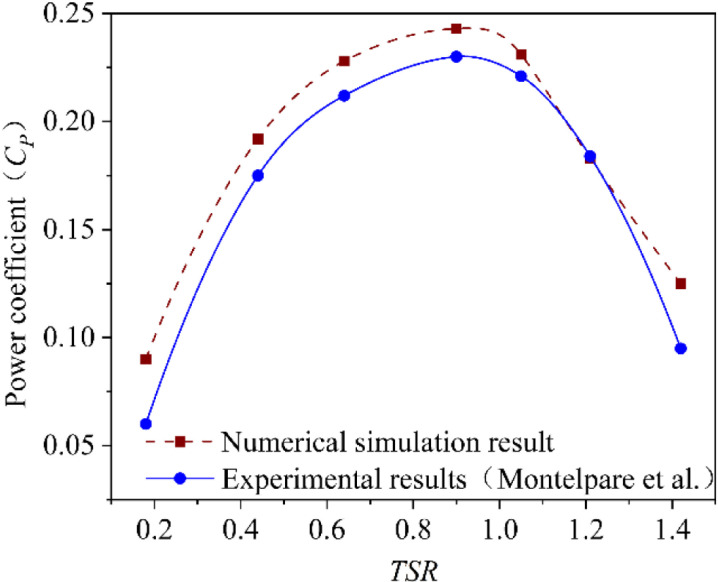
Validation of simulation method.

By comparing CFD analysis using numerical model proposed in this paper with wind tunnel test results obtained by Monteldinger et al [40], validity of the model is verified. Fig 9 shows the numerical model results and the reported experimental results. It can be seen that the numerical simulation results show a similar change trend to the reported experimental results in the area of *TSR* 0.4–1.2. The average error between the two curves is 5.7%. Due to the existence of ideal assumptions in numerical simulation, the results of numerical simulation are usually slightly higher than those of reported experimental results. It can be considered that the numerical simulation method proposed in this paper is suitable for studying the flow characteristics and behavior around rotors.

## 4. Results

The steady and transient of performances of the S-shaped rotor are solved respectively by CFD numerical simulation method, and are compared with that of traditional Savonius rotors.

### 4.1. Steady performance

**Fig 10:**
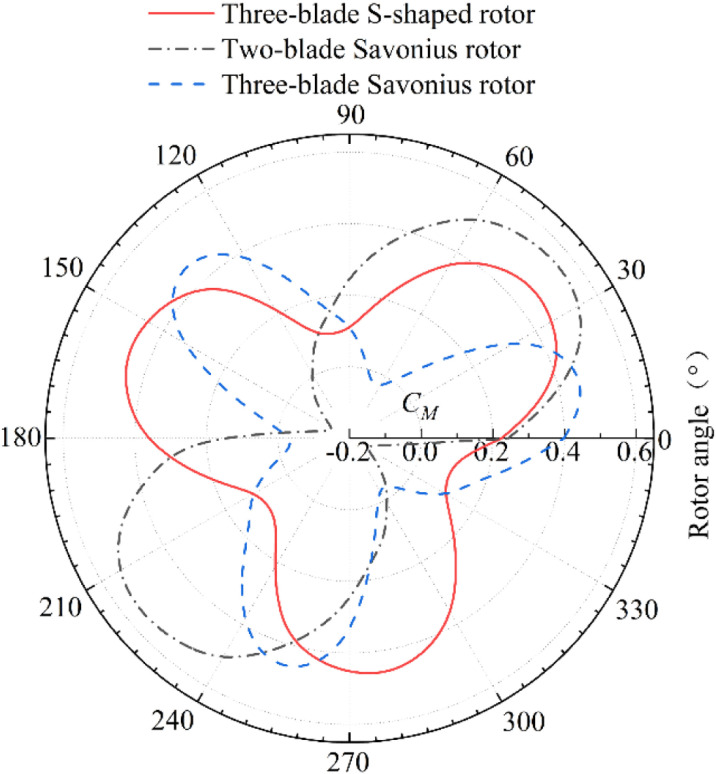
Static torque coefficient curves of three wind rotors.

In order to study steady performance of the wind rotors, two-dimensional steady simulation analysis is carried out under the wind speed condition of 5m/s within the starting wind speed range. Within a rotation period of 120° for the three-blade S-shaped rotor, the position of the rotor is divided at an interval of 10° and the rotor at each attack angle is simulated by Multiple Reference Frame (MRF) method. The rotation period of two-blade Savonius rotor is 180° and 120° for three blades. The results are shown in Fig 10.

The average static torque coefficient and vibration amplitude of the three rotors during operation are shown in Table 5. The three-blade S-shaped rotor has the best driving performance, and its average static torque coefficient is increased by 28.8% compared with the two-blade Savonius rotor. In addition, its vibration amplitude is significantly lower than that of two-blade and three-blade Savonius rotors, which is reduced by 47.1% compared with two-blade Savonius rotor. The smaller the value, the more uniform the stress. It shows that the air impacting on each blade of the S-shaped rotor is relatively uniform during operation. Static torque coefficient of two-blade Savonius rotor is less than 0 when it is in 130° -175° rotation range. The two-blade structure fails to properly guide the airflow at these positions and it causes the airflow to change sharply in the inner cavity region. The negative torque acting on the rotor is greater than the positive torque, causing reverse rotation. It is one of the main reasons for the low power coefficient of Savonius rotors. The vibration amplitude of static torque coefficient curve of the three-blade Savonius rotor is lower than that of the two-blade Savonius rotor and its range of negative torque coefficient is narrow. It indicates that the three-blade arrangement of Savonius rotor can reduce the sharp change of airflow in the inner cavity. However, the static torque coefficient of the three-blade Savonius rotor is low and the driving torque generated by wind is small. The three-blade S-shaped rotor has low vibration amplitude and has no negative torque coefficient range. The average static torque coefficient is higher than the others. It indicates that the S-shaped rotor improves the operating instability of conventional Savonius rotors and increases the starting torque. It has better start-up performance under the condition of low wind speed.

**Table 5:** The average value and vibration amplitude of static torque coefficient.

	S-shaped rotor	Two-blade Savonius rotor	Three-blade Savonius rotor
The average static torque coefficients	0.291	0.226	0.201
Vibration amplitude	0.375	0.709	0.507

### 4.2. Transient performance

Taking the common working wind speed of 10m/s as the simulated working wind speed, the *TSR*s of the rotors are started from 0.2 and set at an interval of 0.2 to analyze the performance changes under different working conditions. Seven rotation cycles are calculated in the numerical simulation, during which the simulation reaches a steady state. The average torque coefficient of the rotor is calculated with the average value of the last rotation cycles and the power coefficient is calculated accordingly.

**Fig 11:**
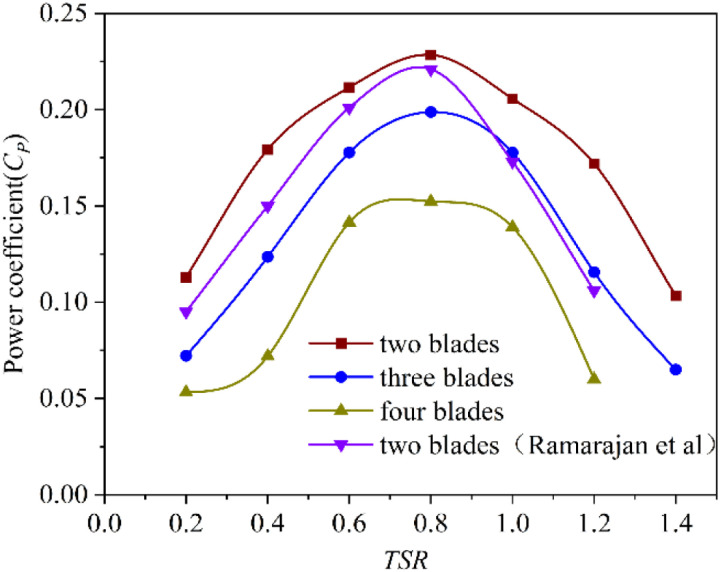
Influence of blade numbers for Savonius rotors.

The performance of Savonius rotors varying with the number of blades is shown in Fig 11. It can be seen that the fluctuation trend of power coefficient of rotors with different blade numbers with *TSR* changes is basically the same. The power coefficient with three different blade numbers reaches the highest value when the *TSR* is 0.8. When the *TSR* is constant, the power coefficient gradually decreases as the number of blades increases. The maximum power coefficient of the four-blade rotor is 0.152. The maximum power coefficient of the three-blade rotor is 0.198. Both of them are less than the maximum power coefficient of the two-blade rotor, which is 0.226. It indicates that the number of blades has a significant impact on the performance of the rotor. Because the rotor structure of the two-blade rotor is simple, the blades do not block each other and can effectively absorb wind energy, so its power coefficient is the highest. As the number of blades increases, the shielding and superposition between adjacent blades increase. It causes a corresponding decrease in the effective stressed area and reduces the wind energy utilization. The performance of the two-blade Savonius rotor studied by Ramarajan et al. [41] is similar to that of the two-blade Savonius rotor in this study. The both power coefficients reach the maximum value when *TSR* is 0.8, which are 0.221 and 0.226 respectively.

**Fig 12:**
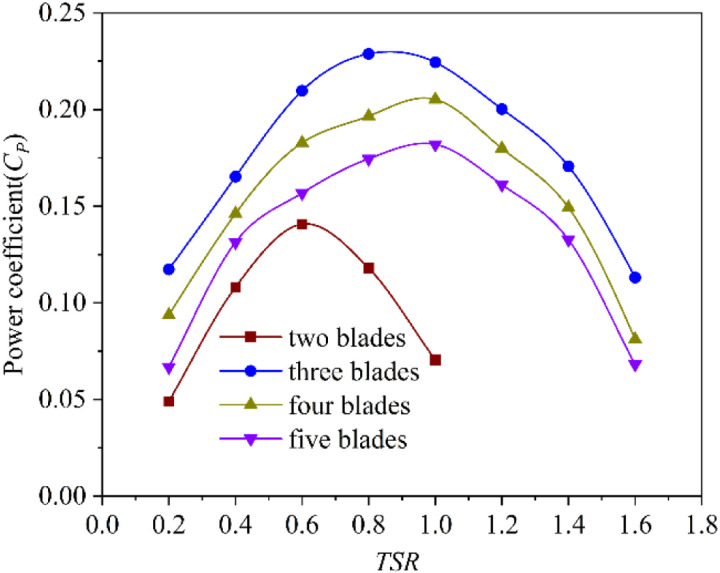
Influence of blade numbers for the S-shaped rotors.

The performance of S-shaped rotors varying with the number of blades is shown in Fig 12. The power coefficient of S-shaped rotors with different number of blades increases first and then decreases with the increase of *TSR*. When the *TSR* is 1.0, the power coefficients of the four-blade and five-blade rotor reach the maximum value, which are 0.205 and 0.182 respectively. When the *TSR* is 0.8, the power coefficient of the three-blade rotor reaches the maximum value of 0.228. When the *TSR* is 0.6, the power coefficient of the two-blade rotor reaches the maximum value of 0.141. It can be seen that with the increase of the number of blades, the maximum power coefficient of the rotor increases first and then decreases, and the power coefficient of the three-blade rotor is the largest. In addition, the *TSR* corresponding to the maximum power coefficient increases first and then decreases with the increase of the number of blades.

**Fig 13:**
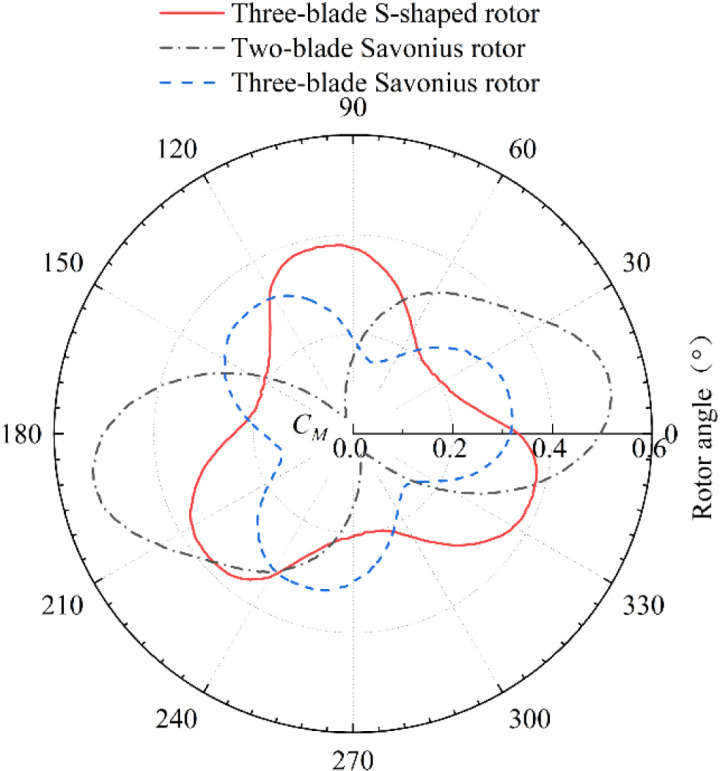
Dynamic torque coefficient curves of three wind rotors.

Figs 11 and 12 show that the two-blade Savonius rotor, three-blade Savonius rotor and three-blade S-shaped rotor all reach the highest power coefficients when the *TSR* is 0.8. Under this condition, the dynamic torque coefficient curve of each rotor is shown in Fig 13. The dynamic torque coefficient curves of the three rotors show similar changing trends. With the change of rotor angle, the dynamic torque coefficient presents a periodic change of decreasing first and then increasing. The average dynamic torque coefficient and vibration amplitude of the three rotors are shown in Table 6. The three-blade S-shaped rotor has the highest average torque coefficient and the best transient output performance. The vibration amplitude of the dynamic torque coefficient curve of the two-blade Savonius rotor is higher than that of the other two rotors. This characteristic indicates that the rotor suffers great impact in the rotation cycle. Therefore, the operating performance is poor and the operation is unstable. The vibration amplitude of the dynamic torque coefficient curve of the three-blade Savonius rotor is much lower than that of two-blade Savonius rotor. It can reduce the impact in the process of operation effectively. However, its average torque coefficient is low in one cycle and the wind energy utilization is lower than that of two-blade rotor. The average dynamic torque coefficient of the three-blade S-shaped rotor is correspondingly higher than that of the three-blade Savonius rotor. The vibration amplitude of its dynamic torque coefficient curve is small, which is reduced by 62.8% compared with the two-blade Savonius rotor. The results show that the operating stability of the rotor is better than that of the two-blade Savonius rotor. The S-shaped blade structure can effectively reduce the oscillation impact of Savonius rotor during operation.

**Table 6:** The average value and vibration amplitude of dynamic torque coefficients.

	S-shaped rotor	Two-blade Savonius rotor	Three-blade Savonius rotor
The average dynamic torque coefficients	0.285	0.282	0.247
Vibration amplitude	0.183	0.492	0.175

The study and analysis of rotors show that the aerodynamic performance of S-shaped rotor with three blades is the best. The average static torque coefficient of the S-shaped rotor is 0.291, which is 28.8% higher than that of the Savonius rotor. The vibration amplitude of its static torque coefficient curve is 0.375, which is reduced by 47.1% compared with the two-blade Savonius rotor. The maximum power coefficient of the three-blade S-shaped rotor is 0.228, which is higher than that of the Savonius rotor. The amplitude of its dynamic torque coefficient curve is 0.183, which is lower than that of the two-blade Savonius rotor.

## 5. Discussion

The comparison of numerical simulation results between S-shaped rotor and Savonius rotor shows that the aerodynamic performance of S-shaped rotor is better than that of Savonius rotor. In order to further explore the reasons for the improvement of aerodynamic performance, the internal flow field characteristics of S-shape rotor and Savonius rotors are studied and analyzed.

### 5.1. Steady performance analysis

**Fig 14:**
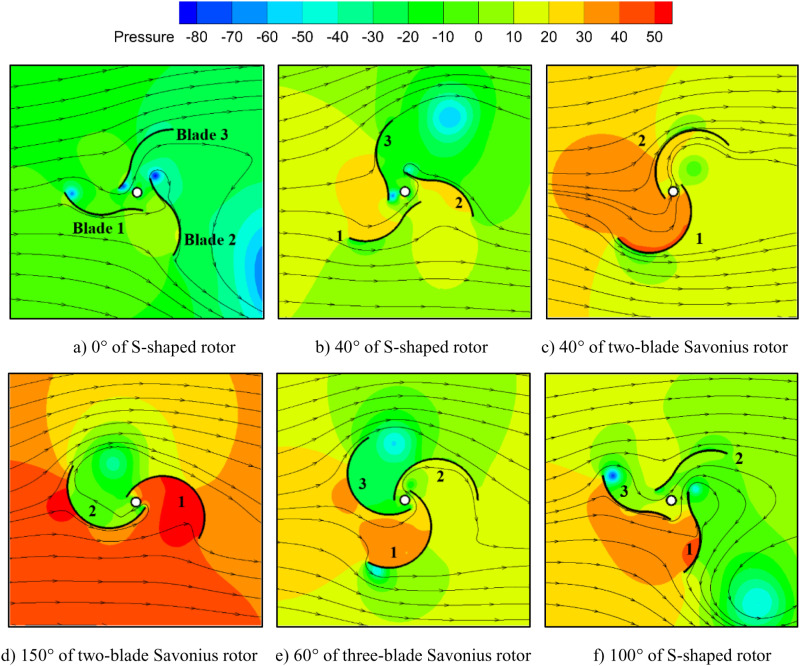
Streamlines at typical positions of the three wind rotors. a) 0° of S-shaped rotor. b) 40° of S-shaped rotor. c) 40° of two-blade Savonius rotor. d) 150° of two-blade Savonius rotor. e) 60° of three-blade Savonius rotor. f) 100° of S-shaped rotor.

Fig 14 shows the streamlines steady flow field (pressure unit Pa) of three wind rotors at some typical positions. Fig 14a shows the internal flow field streamline of the S-shaped rotor at the initial windward position of 0° under static stress. The airflow is divided into two parts through blade 1, one of which is guided by a small concave arc at the inner edge of blade 1 and acts on the big concave arc of blade 2 to form the driving force. The airflow acting on the upper part of blade 1 flows to the leeward side through the overlap area of the inner cavity of the rotor. Because of the three-blade arrangement, the streamline is smoother than that of the two-blade. It reduces the adverse flow and reflux blocking effect occurring in the Savonius rotor. Another part of the airflow acts on the windward side of blade 3 and it hinders the rotation of the wind rotor. However, the windproof effect of blade 3 is reduced due to the change of the attack angle. Therefore, the novel structure can increase the positive driving force, reduce the reverse torque that impedes rotation and improve the wind energy utilization.

Fig 14b shows the position 40° of the S-shaped rotor where static torque coefficient reaches the maximum value. When air flows out the overlap area, small arc convex of blade 2 and inner edge of the blade 3 cooperate with each other to guide the airflow to the suction surface of blade 3. It can reduce the blocking effects of blade 3 at windward side. The concave and convex surfaces of the small circular arc can cooperate with each other to increase the positive driving force of the rotor and guide the airflow gently through the inner cavity area. Fig 14c shows the position 40° of the two-blade Savonius rotor where static torque coefficient reaches the maximum value. Pressure distribution at the windward side of blade 1 is much higher than that of leeward side. The effect of driving force is obvious. At this position, airflow passes through the overlap area smoothly and block effect is not obvious. It indicates that the two-blade Savonius rotor show better performance in some specific locations due to its simple structure. However, as shown in Fig 14d, the two-blade Savonius rotor reaches its minimum static torque coefficient of -0.145 at the position of 150°.At this position, the windward area of the convex surface of the blade 2 is small and the airflow changes sharply in the overlap area. The blocking effect is significant, leading the rotor to generate reverse torque. It obstructs the normal operation of the rotor. Therefore, the defect of two-blade Savonius rotor is also very obvious. The three-blade arrangement is adopted to reduce the sharp change of airflow in the overlap area. As shown in Fig 14e, the static torque coefficient of the three-blade Savonius rotor reaches the minimum value at the position of 60°. At this position, the transition of airflow passing through the overlap area is obviously slowed down and it can reduce energy loss. The static torque coefficient value is -0.036 and it improves the performance of two-blade Savonius rotor. However, due to the semicircle shape of the blade, it is guided by the arc-shaped tip structure of the inner edge of blade 1 and can only impact the inner edge of blade 2 when the airflow flows out of the overlap area. The driving force is close to the central axis and the torque arm is short, so the drive torque is not significantly improved. Fig 14f shows the position 100° of the S-shaped rotor where static torque coefficient reaches the minimum value of 0.093. It shows that the S-shaped blade structure can improve performance of rotor, so that there is no range of negative torque coefficient no reverse rotation. Through the analysis, it can be seen that the rotor with structure of three-blade is more stable in operation than that with structure of two-blade. The structure of three-blade can differentiate the guiding airflow, so that the air impact of the rotor is more uniform during operation. Compared to Savonius blades, the S-shaped blades offer more significant advantages in guiding airflow, allowing the air to sequentially impact multiple blades, which increases forward torque and improves start-up performance of the rotor. The three-blade S-shaped rotor can effectively improve the internal flow field characteristics, overcome the problem of sharp change of airflow in the overlap area, and improve the start-up performance of the rotor.

### 5.2. Transient performance analysis

**Fig 15:**
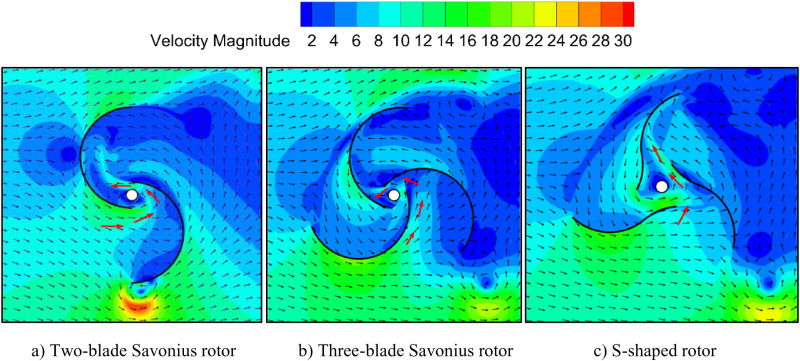
Velocity vectors of the three wind rotors. a) Two-blade Savonius rotor. b) Three-blade Savonius rotor. c) S-shaped rotor.

Fig 15 shows the velocity vector field of three rotors under the condition of wind speed of 10m/s and *TSR* of 0.8. The velocity vector field of the two-blade Savonius rotor is shown in Fig 15a. The airflow enters the overlap area and flow out to the lee side. Since the rotor consists of only two blades, it causes the airflow to change sharply in the overlap area, which affects the wind energy absorption and reduces the smoothness of the rotor operation. Fig 15b shows the velocity vector field of the three-bladed Savonius rotor. The rotor is designed with three blades spaced 60° apart, which smooths the flow through the overlap area. Part of the airflow is directed to the next lee blade. It indicates that this adverse effect can be reduced by increasing the number of blades. However, due to the guidance of the semi-circular blade tip structure, the airflow from the overlap area can only impact the inner edge of the next blade. Since the torque arm is short, the wind energy utilization is not significantly improved. Fig 15c shows the velocity vector field of the three-blade S-shaped rotor. The S-shaped blade adds a small arc to the inner blade side compared with the Savonius blade. By connecting the arc of the blade and distributing the three blades reasonably, the airflow can be guided to impact the concave side of blade at lee side of the rotor.

**Fig 16:**
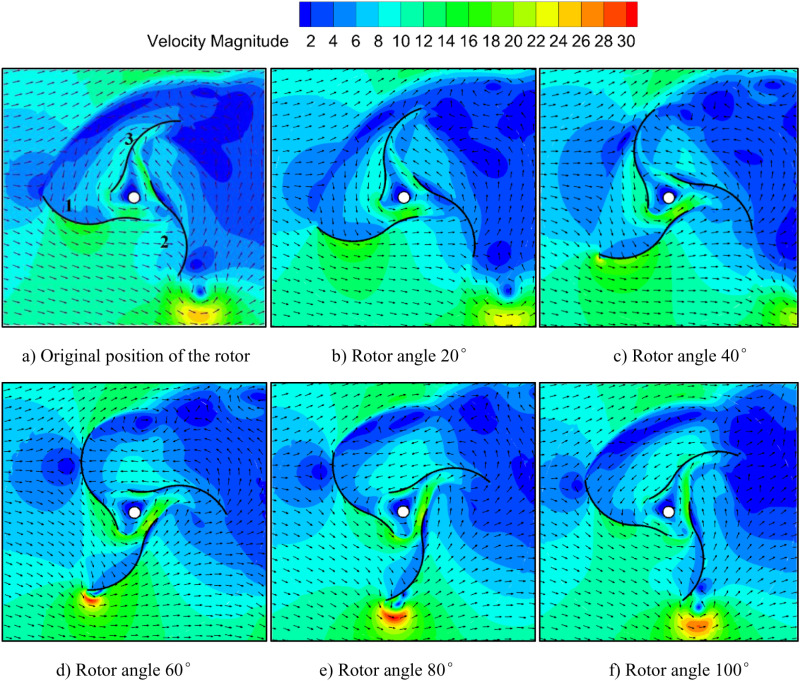
Velocity vectors of the three-blade S-shaped rotor. a) Original position of the rotor. b) Rotor angle 20°. c) Rotor angle 40°. d) Rotor angle 60°. e) Rotor angle 80°. f) Rotor angle 100°.

Simulation results of the velocity vector field of the three-blade S-shaped rotor under the condition of wind speed of 10 m/s and *TSR* of 0.8 are shown in Fig 16. The state of the rotor in Fig 1a is taken as the initial position of the S-shaped rotor. When the rotor is at the initial position of Fig 16a, the airflow over blade 1 is divided into two parts flowing over its convex and concave surfaces. Blade 2 is affected by partial airflow to drive the blade with resistance. At the same time, since the blade 3 is in the return journey and the convex side faces the wind, the resistance torque is formed. However, compared with the two-blade Savonius rotor, the wind resistance is reduced due to the change in attack angle. After driving blade 2, partial airflow passes through the inner cavity to drive blade 3. In Fig 16b, when the rotor rotates 20°, two blades are driven by the wind. At this position, the flow direction is changed by the small arc of blade 2. The concave surface of blade 3 is impacted by the flow. The total driving force is the sum of the two forces of blade 2 and blade 3, and the driving torque is strengthened. In Fig 16c, d, it evolves into a drag-driven blade due to the impact of airflow when blade 1 rotates 60° relative to its initial position. The airflow flows through the concave surface of blade 1 and flows into the overlap area after driving the blade. Then airflow is guided by the small circular arc convex surface of blade 2 to impact the concave surface of blade 3 and form the wind energy reutilization. The effect that airflow passes through the overlap area after driving one blade and drives another blade can also be observed in Fig 16e, f. The S-shaped rotor uses the large and small circular arc in the blade to guide the airflow, so that it impacts the concave and convex surfaces of the blade and strengthens the torque of driving the blade. Because of its unique structure, the three-blade S-shaped rotor can guide the airflow from the windward to the leeward side, improve and smooth the airflow streamline, reduce the blocking effect that often occurs in the inner cavity of the resistance rotor, and improve the aerodynamic performance of the rotor. Moreover, the structure of three-blade allows two of them to absorb wind energy for most of the rotation cycle, expanding the windward area of the blades and enhancing the output performance of the rotor.

The main effect of the structure and configuration of three S-shaped blades is that it enhances the ability that airflow to pass through the overlap area. It is a defect in traditional drag-driven wind rotors of VAWTs. An opening inner cavity is formed by geometric combination of blades. Airflow passing through the rotor can be guided by blades from windward side to leeward side through inner cavity to improve and smoothen streamlined pattern. It can reduce the sharp change of airflow in the overlap area and make the rotor more stable during operation. The structure of S-shaped blades can guide airflow exerting to another blade after it has driven one blade so as to improve starting torque and wind energy utilization. The curvature radius of the blades and angle of installation of blades which form the overlap area have important influence on performance of wind energy reutilization. Small arc of a blade near the overlap area also has important influence on the performance.

The results of flow field analysis show that the two-blade Savonius rotor has a severe airflow transition when the airflow passes through the cavity in the overlap area and it leads to the generation of eddy current and thus reduces the wind energy utilization. The three-blade structure can smooth the airflow, but it will lead to the decrease of wind energy utilization. The large and small circular arc in the blade can guide the airflow, so that it impacts the concave and convex surfaces of the blade and strengthens the torque of driving the blade, when the structure of three S-shaped blades is adopted. Because of its unique structure, the three-blade S-shaped rotor can guide the airflow from the windward to the leeward side, improve and smooth the airflow streamline, reduce the blocking effect that often occurs in the inner cavity of the resistance rotor. The airflow can not only pass through the overlap area smoothly, but also be guided by the small arc of the S-shaped blade to impact the next blade again, thus improving the operating stability and aerodynamic performance of rotor compared with the traditional Savonius rotor. At the same time, the complexity of blade structure does not increase much. Moreover, the complexity of blade structure is not significantly increased. It indicates that the S-shaped rotor can not only overcome the problems of sharp change in the internal flow field of traditional Savonius rotors, but also provide better operating stability and higher wind energy utilization.

## 6. Conclusions

In this paper, a novel three-blade S-shaped rotor is proposed to improve performance of VAWTs. The structure characteristic of the rotor is discussed. The performance is analyzed and compared with traditional Savonius rotors by CFD method. The results show that the rotor has good performance for start-up performance and high power coefficient. There are some conclusions as follow:

1 Based on the advantages and disadvantages of traditional Savonius rotors, a S-shaped rotor is proposed and its structural characteristics are analyzed. The blade is composed of two convex opposite circular arcs. Each blade consists of two arcs whose convex surfaces face each other and are smoothly connected at their ends, and its shape looks like an ‘S’. The rotor is composed of three blades, which are uniformly arranged around the central axis to form a novel S-shaped rotor.
2 The steady and transient performance of the rotor are analyzed and compared with the traditional rotors. The average static torque coefficient of the S-shaped rotor is 0.291, which is 28.8% higher than that of the Savonius rotor. The vibration amplitude of its static torque coefficient curve is 0.375, which is reduced by 47.1% compared with the two-blade Savonius rotor. The maximum power coefficient of the three-blade S-shaped rotor is 0.228, which is higher than that of the Savonius rotor. The amplitude of its dynamic torque coefficient curve is 0.183, which is 62.8% lower than that of the two-blade Savonius rotor. The results show that the novel wind rotor has better performance of start-up performance and high power coefficient than traditional Savonius rotors. It indicates that the S-shaped rotor can not only overcome the problems of sharp change in the internal flow field of traditional Savonius rotors, but also provide better operating stability and higher wind energy utilization.
3 Future works will focus on optimizing the structural parameters of the novel rotor to improve its power coefficient, including installation angle, chord length, height-diameter ratio. A prototype of the optimized S-shaped rotor will also be manufactured and studied by wind tunnel test, and the experimental results will be compared with the simulation results.

### Nomenclature

**Table:** 

*S*	diameter of inscribed circle [m]
*D*	diameter of rotor [m]
*d*	diameter of central axis [m]
*ω*	angular velocity of the rotor [rad/s]
*R*	radius of the rotor [m]
*v*	wind speed [m/s]
*λ*	tip speed ratio
*C*_*p*_	power coefficient
*C*_*M*_	torque coefficient
*P*	power of rotor [W]
*M*	torque [N·m]
*ρ*	air density [kg/m^3^]
*A*	swept area [m^2^]
*G*_*k*_	turbulent kinetic energy term
${\;\;\Gamma \;\;}_{\text{k}}$	effective diffusivity of *k*
${\;\;\Gamma \;\;}_{\;\;\omega \;\;}$	effective diffusivity of *ω*
*Y*_ω_	turbulence of *ω*
*Y*_*k*_	turbulence of *k*
*D*_*ω*_	orthogonal divergent term
**Acronyms**	
HAWTs	horizontal axis wind turbines
VAWTs	vertical axis wind turbines
CFD	computational fluid dynamic
*OL*	overlap ratio
*TSR*	tip speed ratio
SM	sliding mesh
GIL	grid Independent Limit
MRF	multiple reference frame

## Supporting information

S1 File Supporting Information-date. (ZIP)
